# A case series evaluating the serological response of adult asthma patients to the 23-valent pneumococcal polysaccharide vaccine

**DOI:** 10.1186/s13223-017-0200-2

**Published:** 2017-06-07

**Authors:** C. R. Laratta, K. Williams, D. Vethanayagam, M. Ulanova, H. Vliagoftis

**Affiliations:** 1 Pulmonary Research Group, Department of Medicine, University of Alberta, Edmonton, AB Canada; 2 Medical Sciences Division, Northern Ontario School of Medicine, Lakehead University Campus, Thunder Bay, ON Canada; 3 Division of Pulmonary Medicine, Department of Medicine, University of Alberta, Room 3-105 Clinical Sciences Building, 11350 83 Avenue, Edmonton, AB T6G 2G3 Canada

**Keywords:** Invasive pneumococcal disease, Vaccination, Pneumococcal polysaccharide vaccine, Serology, Antibodies, Asthma, Obstructive airways disease, Prevention, Capsular polysaccharide serotypes, Pneumococcal vaccine, PPSV23, Vaccine responsiveness, Corticosteroids

## Abstract

**Background:**

Asthma is an independent risk factor for invasive pneumococcal disease; however, the immune response of adult asthma patients to pneumococcal vaccination is unknown. We explore the serologic response of patients with moderate to severe asthma to the 23-valent pneumococcal polysaccharide vaccine (PPSV23).

**Methods:**

Seventeen moderate to severe adult asthma patients that had not been vaccinated against pneumococcus over the 5 previous years were prospectively recruited from a tertiary care asthma clinic. Serum was analyzed for the presence of antibodies to five capsular polysaccharide (CP) antigens (6B, 9V, 19A, 19F, 23F) before and 4 weeks after PPSV23 vaccination.

**Results:**

There was a wide variability in baseline anti-CP antibody concentrations. Other than for serotype 19A, our patients frequently have baseline anti-CP antibody concentrations below 1 µg/mL (35% for serotype 19F, 41% for serotypes 9V and 23F, and 59% for serotype 6B). All post-vaccination geometric mean antibody concentrations were significantly higher than baseline. In the 31 tests where the baseline antibody concentration was <1 µg/mL, 77.4% had at least a twofold increase post-vaccination. Despite this, a large proportion of post-vaccination anti-CP antibody concentrations remained <1 µg/mL (51.6% of tests). Nine patients had at least one anti-CP antibody concentration <1 µg/mL post-vaccination. There was no difference between these patients and the remaining eight patients in demographic or clinical variables.

**Conclusions:**

Patients with moderate to severe asthma have variable baseline and low post-vaccination antibody concentrations to common CP antigens included in the PPSV23 vaccine. The clinical relevance of these observations remains to be determined since the threshold concentration in adults required for clinical protection from invasive pneumococcal disease is uncertain.

**Electronic supplementary material:**

The online version of this article (doi:10.1186/s13223-017-0200-2) contains supplementary material, which is available to authorized users.

## Background

Invasive pneumococcal disease (IPD) is a common cause of morbidity in adults, occurring at a rate of 9.1 and 9.7 cases per 100,000 people in the North America according to the Centers for Disease Control and Prevention ABCs report [1], and the Public Health Agency of Canada [2], respectively. Vaccinations with pneumococcal capsular polysaccharides (CP) reduce morbidity from IPD [3]. Bigham and colleagues reviewed cases of IPD in British Columbia in 2000, and identified that 89% of the serotypes causing IPD are included into the 23-valent pneumococcal polysaccharide vaccine (PPSV23) [4].

Adults with asthma are at higher risk of developing IPD than the general population [5]. Asthma is a chronic condition affecting as many as 15–20% of the population in developed countries [6]; a population in whom reduction of morbidity and healthcare utilization through preventative measures is an important objective. The Public Health Agency of Canada recognizes asthma “as a high-risk condition warranting vaccination to prevent IPD” and recommends that “adults requiring medical attention for asthma in the last 12 months” should receive one dose of PPSV23 to prevent IPD [7]. These recommendations are similar to other North American guidelines regarding pneumococcal vaccination [8]; however, there remains uncertainty regarding the efficacy of pneumococcal immunization in this population.

Literature on the clinical or serological effect of vaccination in patients with asthma, or on the effects of asthma treatments on vaccination response is limited [9, 10]. In a recent Canadian study, the estimated number of patients with asthma that would need to be vaccinated to prevent one case of IPD may be as low as 246 in low-risk adults and 135 in high-risk adults [11]; however, a number of assumptions had to be used for this analysis as there are limited data specific to this population. Pneumococcal vaccination has been shown to decrease documented pneumococcal pneumonia-related hospitalizations in asthma patients, but have little difference on the risk of pneumonia [12]. Lee et al. [13] demonstrated that children and adolescents with asthma had lower baseline antibody concentrations prior to pneumococcal immunization than healthy children, but post-vaccination geometric mean concentrations (GMC) and the ability to achieve a twofold response were comparable to healthy children. Ohshima and colleagues [14] analyzed serological response to PPSV23 of 40 patients with chronic lung disease including 7 patients with asthma, but did not separately report the results of patients with asthma. Lahood et al. [15] studied pre- and post-vaccination antibody levels for serotypes 3, 7F, 9N, and 14, in a small group of steroid-dependent or non-steroid dependent asthma patients. They found that both groups were able to increase their antibody levels in response to PPSV23; no significant differences between the two groups were noted [15]. Clinical and serological markers of vaccine efficacy in patients with asthma have not yet been established for other respiratory vaccines, such as the influenza vaccine [16]. Limited studies have assessed the impact of high dose inhaled corticosteroid (ICS) therapy or oral corticosteroid therapy on vaccine response in other patient populations [17–19], and there is no data on any vaccine effectiveness with concomitant omalizumab treatment.

As the literature contains very little data on the immunogenicity of vaccination in asthma patients, we have examined baseline antibody concentrations to common pneumococcal serotypes in 17 adult asthma patients, and studied the ability of PPSV23 to increase post-vaccination antibody concentrations. Importantly, we included moderate to severe asthma patients taking high-dose ICS therapy, oral corticosteroid therapy, or biologic therapy, as these patients are at the highest risk of IPD.

## Methods

### Patients and vaccination procedure

We prospectively evaluated the serological response to PPSV23 in a case series of patients with moderate to severe asthma. The study protocol was approved by the University of Alberta Health Research Ethics Board. All patients provided written informed consent. Nineteen patients attending the University of Alberta asthma clinics were enrolled between November 2011 and June 2014. For two enrolled patients, the attending physician questioned the diagnosis of asthma in follow-up visits, and these patients were subsequently excluded from analysis. Fourteen of the 17 patients met criteria for severe asthma as defined per the European Respiratory Society/American Thoracic Society guidelines for severe asthma [20]. Three patients were not using high dose inhaled corticosteroids when recruited and did not meet criteria for severe asthma. However, all these patients were on omalizumab and therefore had a prior diagnosis of moderate to severe asthma, since this is a requirement for treatment with omalizumab in Alberta. Therefore we report all the subjects as having moderate to severe asthma. Six patients had a smoking history. Co-morbid COPD was diagnosed in two of these six patients prior to recruitment. The other four were all under the age of forty, three had a smoking history of only 3–4 pack years and all 4 had no evidence of COPD on pulmonary function testing. One 27 year old non-smoker had a reduced diffusing capacity, but had a normal chest radiograph and no other respiratory diagnosis except from asthma. None of the patients had received the pneumococcal vaccine within the last 5 years. At the time of enrolment, serum was collected for determination of antibodies against pneumococcal CP antigens, and was stored at −70 °C until analysis. Following blood collection, the patient received the PPSV23 vaccine (Pneumovax 23, Merck Sharp & Dohme Corporation, USA) through an intramuscular injection. Three to six weeks later, the serum collection was repeated to follow the antibody response and the sera stored at −70 °C until analysis. Clinical data were obtained through chart review. Pulmonary function testing was performed according to previously published standards [21–23].

### Determination of pneumococcal antibodies

Pre-vaccination and post-vaccination sera were analyzed for antibodies against the following serotype-specific CP antigens: 6B, 9V, 19A, 19F, 23F. Enzyme-linked immunosorbent assay (ELISA) was used to quantify antibodies as per previously published protocols [24]. The lower limit of detection was 0.01 µg/mL. Since there is no consensus with regards to immunological correlates of protection, the following sets of criteria were used to evaluate vaccine response: (a) a significant increase in GMC post-vaccination [25–29]; a twofold increase in antibody concentration [30–36], specifically for ≥2 serotype-specific antigens [30]; or (b) achieving a threshold of 0.35, 1, or 5 µg/mL [25–27, 30, 32, 37, 38].

### Statistical analyses

Data for each patient were coded with a unique identifier. Continuous variables are shown as mean ± standard error of the mean for normally distributed data, and median ±interquartile range (IQR) or 95% confidence interval (CI) for data not normally distributed. The time interval between baseline and post-vaccination serological testing was missing for a single patient, and was included in the analysis as the maximum interval required by any other patient, which was 44 days. Serological measurements are shown as GMC or geometric mean fold rise with two-sided 95% CI. Log-transformed serological data were compared using paired *t* test after normality was confirmed using the Shapiro–Wilk normality test. Continuous variables were compared using the Mann–Whitney *U* test for unpaired data and Wilcoxon signed rank test for paired data. Categorical variables were compared using the Chi square test or Fisher’s exact test. Pre-vaccination and post-vaccination sera GMCs were compared using a two-sample paired t-test after logarithmic transformation. Statistical analyses were performed using GraphPad Prism (GraphPad Prism version 5.00 for Windows, GraphPad Software, San Diego California USA, http://www.graphpad.com).

### Availability of data and materials

The dataset supporting the conclusions of this article is available within this article, with the raw data included in Additional file 1.

## Results

Seventeen patients were included in the analysis, 53% males, mean age 47.4 ± 3.4 years (median 51 years, IQR 33–60 years), with a mean body mass index (BMI) 30.8 ± 1.8 kg/m^2^. Patient demographics are outlined in Table 1. Thirteen patients were on high dose ICS therapy. Of the remaining 4 patients, all were on low to moderate dose ICS therapy with the addition of daily oral corticosteroid therapy (1 patient), or omalizumab (3 patients). No patient was smoking at the time of inclusion; however, 35% of patients had a history of smoking. Two patients had significant non-respiratory co-morbidities, i.e. type II diabetes mellitus and neuromuscular disease. Among 14 patients that had quantitative serum immunoglobulin evaluation, 13 had levels within normal limits, and one had IgA deficiency (0.58 g/L; normal range 0.70–4.00 g/L).

**Table 1:** Patient demographics

	Case series (n = 17)
Age in years	51 (IQR 33–60)
Male gender	9 (53%)
Body mass index (kg/m^2^)	30.8 ± 1.8
History of smoking	6 (35%)
Co-morbid chronic obstructive pulmonary disease	2 (12%)
FEV1^†^ (% predicted)	67.3 ± 4.6
FVC^††^ (% predicted)	90.8 ± 3.4
FEV1/FVC	60.9 ± 2.3
Taking high dose inhaled corticosteroid therapy	13 (76%)
Taking an additional controller agent	16 (94%)
Taking omalizumab therapy	3 (18%)
Taking daily systemic corticosteroid therapy	4 (24%)

^†^ *FEV1* forced expiratory volume in one second

^††^ *FVC* forced vital capacity

The mean time between vaccination and follow-up serological testing was 31.7 ± 2.3 days (minimum and maximum time was 16 and 44 days, respectively). Ten patients had at least one baseline serotype-specific antibody concentration <1 µg/mL (Table 2). For capsular antigens 6B and 23F, the anti-CP antibody concentrations were ≤0.35 µg/mL in 41 and 29% of the patients, respectively (Table 2). After vaccination with PPSV23, there was a significant increase in antibody GMCs against all CP antigens, and all post-vaccination antibody GMCs were >1 µg/mL (Table 3). Among all the serotype-specific antibodies, the lowest geometric mean fold rise was found for the serotype 19A; however, the baseline anti-19A antibody levels were the highest in comparison to other serotypes (i.e. 7.78 µg/mL, 95% CI 4.2–14.4 µg/mL).

**Table 2:** Number (%) of patients with baseline antibody levels below predefined thresholds of 0.35, 1.00, and 5.00 µg/mL

Pneumococcal serotype	Number of patients with baseline anti-CP antibody concentrations failing to achieve specified threshold concentration		
	≤0.35 µg/mL	<1.00 µg/mL	<5.00 µg/mL
6B	7 (41%)	10 (59%)	16 (94%)
9V	2 (12%)	7 (41%)	16 (94%)
19A	0 (0%)	1 (6%)	7 (41%)
19F	3 (18%)	6 (35%)	13 (76%)
23F	5 (29%)	7 (41%)	16 (94%)

**Table 3:** Response to pneumococcal vaccination in the overall cohort

Pneumococcal serotype	Serum samples	Anti-CP GMC^†^ (95% CI) (µg/mL)	Mean fold rise (95% CI)
6B	Baseline	0.54 (0.21–1.38)	–
	Post-vaccination	2.13 (0.72–6.28)**	3.96 (1.70–9.22)
9V	Baseline	1.19 (0.64–2.22)	–
	Post-vaccination	4.25 (2.19–8.24)***	3.57 (1.84–6.93)
19A	Baseline	7.78 (4.2–14.4)	–
	Post-vaccination	15.34 (8.39–28.05)**	1.97 (1.26–3.09)
19F	Baseline	1.70 (0.64–4.56)	–
	Post-vaccination	7.76 (3.26–18.46)***	4.56 (2.26–9.21)
23F	Baseline	0.94 (0.43–2.08)	–
	Post-vaccination	3.81 (1.56–9.30)****	4.03 (2.35–6.92)

^†^ *Anti-CP GMC* anti-capsular polysaccharide geometric mean concentration

** p < 0.01, *** p < 0.001, **** p < 0.0001

**Fig. 1:**
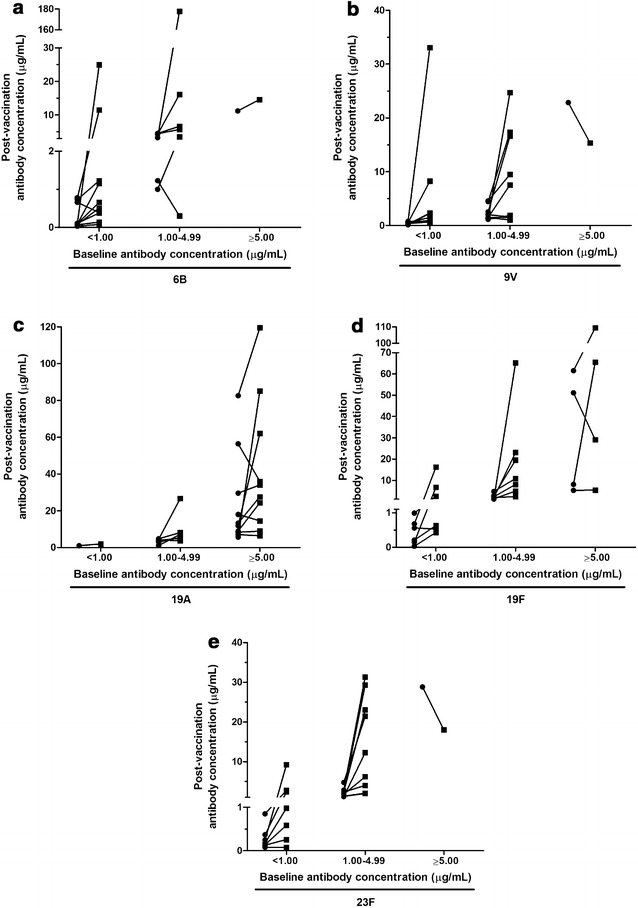
Individual antibody responses to vaccination for capsular antigens **a** 6B, **b** 9V, **c** 19A, **d** 19F, **e** 23F. The patients are displayed in three categories: those who started with a baseline concentration <1 µg/mL, between 1 and 4.99 µg/mL, and ≥5 µg/mL

**Fig. 2:**
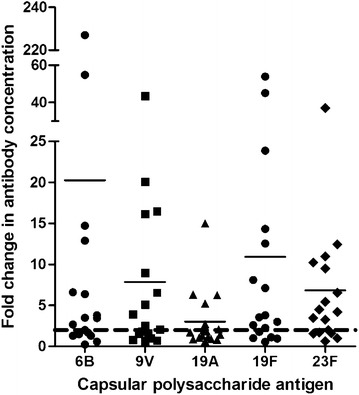
Fold change in serotype-specific antibody levels post-vaccination. Data for individual patients and mean fold change is shown. The interrupted line identifies the twofold threshold increase in antibody concentration

The overall substantial increase in GMC was a result of a highly variable individual response to vaccination. Individual changes in anti-CP antibody concentrations post-vaccination for each patient are outlined in Fig. 1, organized by the baseline antibody levels of <1.00, 1.00–4.99, or ≥5.00 µg/mL. As can be seen in Fig. 1, the post-vaccination anti-CP antibody concentrations increased in some patients and decreased in others, and these changes were not consistent between serotypes. Fold change in serotype-specific antibody concentrations for each patient is shown in Fig. 2.

Although we tried to collect post vaccination serum 3–6 weeks after vaccination, we had some 6 subjects that came back outside this interval. In order to ensure that our findings are not accounted for by either a short or long interval between the vaccination and follow-up serology, we analyzed the data separately for those with follow-up blood collection less than 3 weeks or more than 6 weeks after vaccination. Notably, the three patients who had blood for determination of antibody concentrations collected less than 3 weeks (3 patients) or more that 6 weeks (3 patients) after vaccination all achieved a twofold increase to at least two antigens, and only one had antibody concentrations <1.00 μg/mL in the post-vaccination serum collection (capsular antigens 6B and 23F). The data for these three patients is shown in Additional file 1 under patient identifiers 9, 10, 12, 13, 16 and 17. Excluding these patients from the analyses did not change the results (data not shown).

The serological response of asthma patients to the PPSV23 was variable among patients as well as among serotype-specific antibodies within individual patients. When a twofold rise in concentration was used as a marker of response, the majority of the patients responded to the vaccination, i.e. 13 out of the 17 patients (76.5%) mounted a twofold response to ≥2 antigens. Very high baseline antibody concentrations may have contributed to the inability of some patients to achieve a twofold rise in post-vaccination antibody concentrations. For example, in one patient, the anti-19A CP antibody level increased from 82.55 to 119.55 µg/mL, which is a 1.45-fold rise. Four patients mounted a twofold antibody increase to 0–1 antigens, 4 had a twofold response to 4 antigens, and 4 patients had a twofold response to all 5 antigens.

When achievement of a certain threshold concentration was used to determine the response to vaccination, the results were again highly variable. In Table 4, we summarize the antibody responses of patients who had a baseline antibody concentration below 1 µg/mL. Ten patients had at least one baseline anti-CP antibody concentration <1 µg/mL. After vaccination, nine patients had at least one anti-CP antibody concentration <1 µg/mL. Eight of the 9 patients in this group had a baseline antibody concentration <1 µg/mL, but the ninth patient had an anti-CP antibody concentration <1 µg/mL post-vaccination only (1.23 µg/mL at baseline and 0.30 µg/mL post-vaccination for the capsular antigen 6B). In these 9 patients, post-vaccination levels <1 µg/mL were detected against serotype 6B (7 patients), 9V (2 patients), 19F (3 patients), and 23F (4 patients). In the individuals with a baseline antibody concentration <1 µg/mL, only 19% of the serotype-specific antibodies increased to ≥5 µg/mL post-vaccination indicating that PPSV23 was able to induce a robust vaccine response in a minority of these patients.

**Table 4:** Response to vaccination of patients with baseline antibody concentrations of <1 µg/mL

Pneumococcal serotype	Twofold response to vaccination	Post-vaccination anti-CP^†^ antibody concentration		
		≥0.35 µg/mL	≥1.00 µg/mL	≥5.00 µg/mL
6B (n = 10)	8	7	3	1
9V (n = 7)	5	7	5	2
19A (n = 1)	0	1	1	0
19F (n = 6)	5	5	3	2
23F (n = 7)	6	6	3	1

^†^ *CP* capsular polysaccharide

Contingency analysis and logistical regression were used to determine whether any patient-related factors were associated with response to vaccination. There was no predilection for gender or age to affect response to immunization with PPSV23 within our case series; however, all patients included were younger than 65 years. The phenotype of asthma including timing of onset, or atopy as documented on skin prick testing, peripheral eosinophil count, or IgE concentration was not associated with a response to vaccination. Obesity was not associated with vaccine response either. Treatment with biologics, high dose inhaled corticosteroid therapy, or systemic steroids, was not associated with the ability to mount a response to vaccination (data not shown). Similarly, there was no significant association between the presence of daily corticosteroid use or steroid burst in the last year with either the inability to mount a ≥twofold response in antibody concentrations to at least two capsular antigens [relative risk (RR) 0.70, 95% CI 0.13–3.85; p = 1.0], or to increase in the post-vaccination anti-CP antibody concentration to ≥1 µg/mL (RR 0.42, 95% CI 0.15–1.21; p = 0.15).

## Discussion

Preventative strategies to reduce the impact of IPD in patients with asthma may have substantial impact on patient morbidity, with secondary gains in the form of reduced health care utilization and a decreased need for health care resources. This study presents pre- and post-vaccination antibody concentrations of adult patients with moderate to severe asthma immunized with PPSV23. In this population, baseline anti-CP GMCs are highly variable among both individuals and serotypes, consistent with previous findings in the general population, the elderly, and adults with chronic or immunosuppressive medical conditions [14, 26, 27, 31, 32, 34–36, 38–42]. Comparable baseline serologic data are limited to one study by Lahood et al. [15], that also reports highly variable baseline antibody concentrations in asthma patients. Of the serotype-specific antibodies included in our analysis, the highest baseline concentrations were found against serotype 19A, raising the possibility that asthma patients in our population may be exposed to this serotype in the community. Serotype 19A has been implicated as a cause of “replacement disease” following the introduction of 7-valent pneumococcal protein-conjugate vaccine (PCV) [43, 44].

A response to vaccination has been defined with a variety of methods in the literature, such as the ability to achieve a significant rise in GMC, fold rise in antibody concentration, or an antibody concentration threshold that is considered protective; however, interpretation of these data is complicated in that immunological correlates of protection have not been established for adults [45]. Antibody thresholds conferring clinical protection are likely to be serotype specific, as suggested from studies in children [46]. Published data on adult immunization with PPSV23 are of limited comparative value as the subjects are often populations with high prevalence of IPD, such as subjects with asplenia [36, 37]. The knowledge on serologic responses to PPSV23 in patients with asthma is very limited, with only two small studies available [13, 15]. Lee et al. [13] report a twofold rise in antibodies in 41.6–87.5% of their 24 pediatric asthma patients after vaccination with PPSV23, which is comparable to our data. Absolute antibody concentrations from this study cannot be compared to our results because they were expressed as % of reference serum [13]. Lahood et al. [15] performed a study comparing 14 adult asthma patients on prednisone to 14 adult asthma patients not taking prednisone, and reported increases in antibody concentrations 4 weeks after vaccination with PPSV23 [15]. As they report post-vaccination antibody concentrations measured by a different immunoassay, their data cannot be directly compared to our results.

Our study suggests that patients with asthma may remain at risk of developing IPD after receiving vaccination with PPSV23, as they may not achieve an adequate antibody concentration threshold that confers clinical protection. Indeed, although the majority of our patients (13 out of 17) responded to immunization with a twofold increase in antibody concentrations to at least 2 antigens out of 5, 9 patients had at least one post-vaccination antibody concentration <1 µg/mL. In comparison, in an earlier study, 4–6 weeks post-PPSV23 immunization, most of healthy adults had the concentrations of serotype-specific pneumococcal antibody >1 µg/mL, i.e. 73 and 82% against the serotype 6B, 88 and 83% against 19F, and 85 and 85% against 23F, for subjects of 20–69 and ≥70 years of age, respectively [47].

Although interpretation of these findings is complicated by the fact that immunologic correlates of protection against IPD in adults are not well described, it has been demonstrated that non-immunocompromised 50–85 year old subjects who developed culture-verified pneumococcal pneumonia post PPSV immunization, failed to achieve a >1 µg/mL antibody concentration threshold for the infecting serotype [48]. These data support our suggestion that 9 out of 17 asthma patients in our study may remain at risk of pneumococcal infection.

Although this is a pilot study with a small number of participants, our report intends to draw attention to an urgent need to improve pneumococcal immunity in adults with moderate to severe asthma who are highly susceptible to IPD. In this category of patients, immunization with the pneumococcal polysaccharide vaccine may be insufficient for inducing protective immunity against *S. pneumoniae*. As an option, immunization with the conjugate protein pneumococcal vaccine (PCV13) should be considered because it may confer better immune response to the capsular polysaccharide antigens in certain categories of immunocompromised adults [49–51]. In Canada, PCV13 has recently been approved for immunization of immunocompromised adults, including subjects with primary immunodeficiency, malignant neoplasms, hematopoietic stem cell, solid organ, or islet transplantation, treatment with immunosuppressive therapy, and HIV-infection (Canadian Immunization Guide). However, with regards to using PCV13 for immunization of adults it is important to consider that it contains only 13 capsular polysaccharides as compared to 23 in PPSV23, and was designed to primarily target the most important pediatric *S. pneumoniae* serotypes. More research is needed to develop optimal immunization strategy for asthma patients, in particular, to determine the prevalence of various *S. pneumoniae* serotypes in IPD and pneumococcal pneumonia in this group of patients and to address the impact of various asthma treatments on immune response to pneumococcal immunization.

There are several limitations to this study, including a small number of asthma patients, and a risk of selection bias, given that we did not enrol consecutive patients from our asthma clinic. In addition, an interval between pre- and post-immunization samples varied among the participants although this unlikely affected our results, considering that the half-life of IgG is approximately 4 weeks [52], and the majority of studies of the response to PPSV23 vaccination in various populations report serologic testing between 25 and 46 days post-immunization [25, 30, 38, 51, 53, 54]. In addition, exclusion of participants with short or long intervals between pre- and post-vaccination evaluation of antibody concentrations did not change the results of our analysis. In our study, we did not address the functional antibody activity, which would require an opsonophagocytic assay. Finally, the lack of a healthy control group for direct comparison of immune responses to immunization is a limitation of this study. However, the response of healthy adults to PPSV23 has been previously reported by several other studies [55]. One of the advantages of our study is that it is prospective in nature. This study is also important in that it explores vaccination in a population that may derive significant benefit from optimized protection against IPD, and in whom optimization of preventative therapy may result in large gains in terms of health-care utilization and resource allocation.

## Conclusions

While many adult patients with asthma are able to generate at least a twofold increase in antibody concentrations in response to immunization, the majority of patients had at least one post-vaccination anti-CP antibody concentration <1 µg/mL, and may not be achieving a threshold associated with clinical protection against IPD. The clinical relevance of these observations remains to be determined since the threshold concentration in adults required for clinical protection from IPD is unknown. Further research is required to determine if PPSV23 or other pneumococcal vaccines are capable of inducing adequate post-vaccination anti-CP antibody concentrations that confer clinical protection. The timing of vaccination in this patient population should also be studied, as high baseline antibody concentrations to serotype 19A in our population may indicate frequent exposure to this serotype. Further research into these questions is required in order to optimize recommendations to prevent IPD in this at-risk population.

## Additional file

**Additional file 1.** Raw data of anti-serotype antibody concentrations before and after vaccination for all subjects participating in the study.

## Abbreviations

IPD: invasive pneumococcal disease
CP: capsular polysaccharide
PPSV23: 23-valent pneumococcal polysaccharide vaccine
GMC: geometric mean concentration
IQR: interquartile range
CI: confidence interval
BMI: body mass index
FEV1: forced expiratory volume in one second
FVC: forced vital capacity
PCV: pneumococcal protein-conjugate vaccine
