# Supplementary material for: A case series evaluating the serological response of adult asthma patients to the 23-valent pneumococcal polysaccharide vaccine
Source: Allergy Asthma Clin Immunol. 2017 Jun 7;13:27. doi: 10.1186/s13223-017-0200-2 (PMC5463404; doi:10.1186/s13223-017-0200-2)
Supplement: Supplementary file 1 — Additional file 1. Raw data of anti-serotype antibody concentrations before and after vaccination for all subjects participating in the study. [file 13223_2017_200_MOESM1_ESM.docx]

Additional file 1: Raw data of antibody concentrations

Table S1: Baseline anti-capsular polysaccharide antibody concentrations

| Patient identifier | Capsular Polysaccharide Serotype | | | | |
| --- | --- | --- | --- | --- | --- |
|  | 6B | 9V | 19A | 19F | 23F |
| 1 | 0.10 | 2.01 | 3.84 | 2.03 | 1.27 |
| 2 | 0.78 | 1.48 | 8.96 | 0.68 | 0.37 |
| 3 | 1.23 | 1.45 | 18.00 | 2.27 | 1.29 |
| 4 | 4.43 | 22.85 | 56.40 | 51.12 | 28.83 |
| 5 | 0.11 | 0.14 | 0.99 | 0.03 | 0.25 |
| 6 | 0.07 | 0.37 | 4.26 | 0.56 | 0.13 |
| 7 | 0.08 | 0.59 | 4.54 | 1.84 | 2.36 |
| 8 | 0.73 | 1.50 | 8.36 | 5.31 | 2.36 |
| 9 | 0.09 | 0.26 | 3.00 | 0.99 | 0.18 |
| 10 | 0.11 | 0.60 | 7.01 | 0.21 | 0.14 |
| 11 | 3.24 | 1.23 | 13.29 | 2.75 | 1.20 |
| 12 | 11.22 | 2.53 | 82.55 | 61.56 | 2.43 |
| 13 | 0.03 | 0.50 | 1.15 | 0.15 | 0.08 |
| 14 | 0.66 | 0.76 | 5.68 | 1.21 | 0.85 |
| 15 | 1.00 | 1.15 | 4.76 | 1.31 | 2.85 |
| 16 | 4.5 | 4.57 | 29.58 | 4.78 | 4.76 |
| 17 | 4.26 | 4.43 | 11.85 | 8.10 | 1.78 |

Table S2: Post-vaccination anti-capsular polysaccharide antibody concentrations

| Patient identifier | Number of days between vaccination and repeat serology | Capsular Polysaccharide Serotype | | | | |
| --- | --- | --- | --- | --- | --- | --- |
|  |  | 6B | 9V | 19A | 19F | 23F |
| 1 | 40 | 0.66 | 1.59 | 5.06 | 2.28 | 1.96 |
| 2 | 28 | 11.48 | 7.56 | 24.37 | 16.23 | 2.42 |
| 3 | 38 | 0.30 | 1.05 | 14.54 | 8.07 | 2.06 |
| 4 | 37 | 5.72 | 15.34 | 36.03 | 29.10 | 18.02 |
| 5 | 37 | 24.97 | 2.26 | 1.97 | 0.43 | 9.26 |
| 6 | 34 | 0.14 | 0.68 | 3.60 | 0.53 | 0.26 |
| 7 | 28 | 0.51 | 1.48 | 28.68 | 23.08 | 29.33 |
| 8 | 35 | 1.23 | 1.51 | 8.93 | 5.38 | 4.00 |
| 9 | 44 | 1.16 | 2.33 | 5.49 | 2.58 | 0.98 |
| 10 | 44 | 0.38 | 0.91 | 6.39 | 0.63 | 0.59 |
| 11 | 31 | 177.76 | 24.72 | 27.6 | 19.57 | 12.27 |
| 12 | 18 | 14.61 | 16.6 | 119.55 | 109.36 | 23.07 |
| 13 | 16 | 0.08 | 8.23 | 7.20 | 6.77 | 0.08 |
| 14 | 23 | 0.39 | 33.06 | 85.21 | 65.26 | 2.78 |
| 15 | 24 | 3.49 | 1.87 | 8.17 | 4.98 | 31.33 |
| 16 | 44 | 6.66 | 9.49 | 34.04 | 10.87 | 21.43 |
| 17 | 18 | 16.14 | 17.34 | 62.13 | 65.58 | 6.20 |
